# Using a Targeted Proteomics Chip to Explore Pathophysiological Pathways for Incident Diabetes– The Malmö Preventive Project

**DOI:** 10.1038/s41598-018-36512-y

**Published:** 2019-01-22

**Authors:** John Molvin, Manan Pareek, Amra Jujic, Olle Melander, Lennart Råstam, Ulf Lindblad, Bledar Daka, Margrét Leósdóttir, Peter M. Nilsson, Michael H. Olsen, Martin Magnusson

**Affiliations:** 1 Department of Clinical Sciences, Lund University, Clinical Research Center, Malmö, Sweden; 2 Department of Cardiology, Skåne University Hospital Malmö, Malmö, Sweden; 3 Cardiology Section, Department of Internal Medicine, Holbæk Hospital, Holbæk, Denmark; 4 Brigham and Women’s Hospital Heart & Vascular Center, Harvard Medical School, Boston, Massachusetts USA; 5 Department of Internal Medicine, Skåne University Hospital, Malmö, Sweden; 6 Institute of Medicine, Department of Public Health and Community Medicine, Sahlgrenska Academy, University of Gothenburg, Gothenburg, Sweden; 7 Centre for Individualized Medicine in Arterial Diseases (CIMA), Odense University Hospital, University of Southern Denmark, Odense, Denmark; 8 Wallenberg Center for Molecular Medicine, Lund University, Lund, Sweden

## Abstract

Multiplex proteomic platforms provide excellent tools for investigating associations between multiple proteins and disease (e.g., diabetes) with possible prognostic, diagnostic, and therapeutic implications. In this study our aim was to explore novel pathophysiological pathways by examining 92 proteins and their association with incident diabetes in a population-based cohort (146 cases of diabetes versus 880 controls) followed over 8 years. After adjusting for traditional risk factors, we identified seven proteins associated with incident diabetes. Four proteins (*Scavenger receptor cysteine rich type 1 protein M130*, *Fatty acid binding protein 4, Plasminogen activator inhibitor 1* and *Insulin-like growth factor-binding protein 2*) with a previously established association with incident diabetes and 3 proteins (*Cathepsin D*, *Galectin-4, Paraoxonase type 3*) with a novel association with incident diabetes. *Galectin-4*, with an increased risk of diabetes, and *Paraoxonase type 3*, with a decreased risk of diabetes, remained significantly associated with incident diabetes after adjusting for plasma glucose, implying a glucose independent association with diabetes.

## Introduction

The worldwide prevalence of type 2 diabetes has risen steadily from 108 million in 1980 to 422 million in 2014 and constitutes a major threat to public health through increased morbidity and mortality^1^. Almost half of all deaths attributable to hyperglycemia occur before the age of 70 years, highlighting the need for early identification and lifestyle interventions of high-risk individuals as well as identifying novel therapeutic targets^2^. Although often simplified to a combination of insulin resistance and insulin deficiency, much remains to be explored regarding the complex pathogenic processes underlying the disease. This creates a rationale for applying a multi-system approach, including the exploration of pathophysiological pathways that may have diagnostic, prognostic, and therapeutic implications.

The recently developed proximity extension assay technology^3^ has enabled simultaneous analyses of large sets of proteins in small biological sample volumes. We used such an immunoassay designed to analyze 92 proteins with proposed involvement in inflammation / immunity, cardiovascular disease, and metabolism, in order to explore potential pathophysiological pathways for incident diabetes in a population-based cohort.

## Materials and Methods

### Study sample

During 1974–1992, specific birth cohorts between 1921 and 1949 of inhabitants in Malmö, Sweden, were invited to participate in a large cohort study, i.e., the *Malmö Preventive Project (MPP)*, with a total of 33,346 individuals attending (attendance rate 71%). Re-examination of 18,238 *MPP* survivors, who were still residing in the Malmö area, the *MPP Re-Examination Study (MPP-RES)*, was conducted during 2002–2006 (attendance rate 72%). In a subsample of 1,792 participants, echocardiography was performed. These subjects were randomly selected from groups defined by glucometabolic status: normal fasting glucose, impaired fasting glucose, new onset diabetes and prevalent diabetes, with oversampling in the groups with glucometabolic disturbances to ensure numerical balance, as described previously^4^. The reason for this oversampling was to ensure sufficient numbers in each group as the study originally was designed to investigate myocardial structure and function in elderly subjects in relation to their glucometabolism. Data on lifestyle and medical history were obtained through a self-administered questionnaire. Physical activity was self-reported and categorized into 4 levels from sedentary lifestyle to physically active at a great extent. Height and weight were measured and body mass index (BMI, kg/m^2^) subsequently calculated. Blood pressure was measured twice in the supine position after 10 minutes of rest, and blood samples were drawn after an overnight fast and stored at −80 °C. Hypertension was defined as systolic blood pressure (SBP) >140 mmHg and diastolic blood pressure (DBP) >90 mmHg or the use of anti-hypertensive medication. Plasma samples from a total of 1,737 individuals from this subsample were successfully analyzed with the Olink Proseek Multiplex CVD III 96 × 96 proximity extension assay. Patients with missing covariates at baseline (n = 30) and prevalent diabetes (n = 681) were excluded, resulting in 1026 eligible subjects for the main analyses of incident diabetes.

All participants signed a written informed consent form before entering *MPP-RES*. The study was approved by The Regional Ethical Review board at Lund University, Sweden (LU 244-02) and complied with the Helsinki Declaration.

### Proteomic Profiling

Plasma levels of proteins were analyzed by the Proximity Extension Assay (PEA) technique using the Proseek Multiplex CVD III 96 × 96 reagents kit (Olink Bioscience, Uppsala, Sweden) which uses two oligonucleotide-labeled highly specific antibodies to bind to each target protein, which allows the formation of a polymerase chain reaction sequence that can then be detected and quantified^3^. The CVD III panel, published in several well-renowned journals^5–7^, consists of ninety-two proteins, carefully selected by leading experts in the field, with either established or proposed association with cardiovascular disease, inflammation and metabolism. The CVD I panel partially overlapping with the CVD III panel has previously been used to explore potential biomarkers for insulin resistance^8^ but no similar studies have been performed using the CVD III panel. The CVD III panel was also recently used for replication in a publication describing the genomic atlas of the human plasma proteome^9^. All data are presented as arbitrary units. One protein was below detectable limits in >15% samples (N-terminal pro-brain natriuretic peptide (Nt-proBNP)). Across all 92 assays, the mean intra-assay and inter-assay variations were observed to be 8.1% and 11.4%, respectively. Validation data and coefficients of variance for all proteins can be found in the online supplemental material (Validation data CVD III) and further technical information about the assays are available on the Olink homepage (http://www.olink.com).

### Laboratory Assays

All fasting analyses (plasma glucose, serum high-density lipoprotein (HDL),, and serum triglycerides (TG)) were performed at the Department of Clinical Chemistry, Malmö University Hospital, attached to a national standardization and quality control system (Beckman Coulter LX20, Beckman Coulter Inc., Brea, USA). Plasma cystatin C was analysed with an automated particle-enhanced immunoturbidimetric method, using reagents from DakoCytomation (Glostrup, Denmark).

### Classification of prevalent and incident diabetes in MPP-RES

Prevalent diabetes at baseline was defined as a self-reported physician diagnosis of diabetes, use of antidiabetic medication, a diagnosis of diabetes in any of the local or national diabetes registries prior to study entry, or two separate fasting plasma glucose measurements of ≥7.0 mmol/L when available. Incident diabetes was retrieved through record linkage of the Swedish personal identification number with national and regional registries as follows: The Malmö HbA1_c_ Register that analyzed all HbA1_c_ samples at the Department of Clinical Chemistry obtained in institutional and non-institutional care in Malmö from 1988 and onwards^10^; The Swedish National Diabetes Register^11^; The Regional Diabetes 2000 Register of the Skåne Region^12^; The Swedish National Patient Register covering all somatic and psychiatric hospital discharges and hospital based outpatient care^13^; The Swedish Cause-of-Death Register^14^; and The Swedish Prescribed Drug Register (prescription of anti-diabetic medication)^15^. Type of diabetes was not specified from all registries but given the mean age of the study population and since all prevalent cases of diabetes were excluded, it is reasonable to assume that an absolutely overwhelming majority of the incident cases of diabetes were type 2 diabetes.

### Statistical Analysis

Non-normally distributed variables (all 91 proteins, TG, HDL, glucose and cystatin C) were ln-transformed prior to analysis. Cox proportional-hazards regression models and Harrell’s concordance index (C-index)^16^ were used to calculate hazard ratios (HRs) for incident diabetes per standard deviation (SD) of change of log-transformed values in age- and sex-adjusted models (model 1). Proportional hazard assumption was tested using Schoenfeld residuals. Only proteins that remained significant after Bonferroni correction (0.05/91 = 5.5 × 10^−4^) in model 1 were further tested in the multivariable Cox regression model and Harrell’s C-index (model 2), which was adjusted for age, sex, BMI, hypertension, antihypertensive treatment, TG, HDL, cystatin C and physical activity and furthermore in model 3 (entering fasting plasma glucose at baseline on top of model 2). The proteins associated with incident diabetes in model 1 were also tested for association with prevalent diabetes with binary logistic regression in models 1, 2 and 3.

All analyses were performed using SPSS Statistics version 22.0 (IBM, Armonk, New York, USA).

## Results

Baseline characteristics of subjects with (n = 681) and without (n = 1026) prevalent diabetes are listed in Table 1. Subjects with prevalent diabetes at baseline had higher TG and lower HDL levels, higher BMI, increased prevalence of hypertension, and worse renal function as measured by cystatin C (Table 1). Baseline characteristics of the 1026 subjects examined for incident diabetes are listed in Table 2. Of these, 146 developed diabetes during the median follow-up time of 8.0 years (interquartile range 12 years). Subjects with incident diabetes were more often male, had higher blood pressure, TG and lower HDL levels, as well as greater BMI at baseline, compared with those who did not develop diabetes.

**Table 1:** Baseline Characteristics of Study Participants with and without Prevalent Diabetes at Baseline examination.

	All subjects (n = 1707)	Subjects without prevalent diabetes (n = 1026)	Subjects with prevalent diabetes (n = 681)	p-value
Age (years)	67.4 (±6.0)	66.9 (±6.1)	68.1 (±5.9)	<0.001
Sex (% female)	498 (29.1)	331 (32.2)	167 (24.4)	0.001
BMI	28.3 (±4.3)	27.4 (±3.9)	29.8 (±4.6)	<0.001
SBP (mmHg)	146.9 (±19.8)	145.3 (±19.2)	149.1 (±20.6)	<0.001
HT (%)	1069 (62.6)	570 (55.6)	499 (73.3)	<0.001
FPG (mmol/l)	6.2 (5.6–7.4)	5.8 (5.3–6.2)	7.8 (7.1–9.2)	<0.001
TG (mmol/l)	1.3 (0.9–1.8)	1.1 (0.8–1.6)	1.5 (1.0–2.0)	<0.001
HDL (mmol/l)	1.3 (1.0–1.5)	1.3 (1.1–1.6)	1.2 (1.0–1.4)	<0.001
Cystatin C (mg/l)	1.06 (0.95–1.20)	1.05 (0.95–1.19)	1.08 (0.95–1.24)	0.002

BMI; body mass index, SBP; systolic blood pressure; DBP; diastolic blood pressure, HT; hypertension, FPG; fasting plasma glucose, TG; triglycerides, HDL; high-density lipoprotein cholesterol. Values are displayed as means (±standard deviation) or, for skewed variables, medians and interquartile (25–75) range.

**Table 2:** Baseline Characteristics of Study Participants with and without Incident Diabetes at Baseline Examination.

	All subjects (n = 1026)	Subjects without incident diabetes (n = 880)	Subjects with incident diabetes (n = 146)	p-value
Age (years)	66.9 (±6.1)	66.9 (±6.1)	66.7 (±5.8)	0.071
Sex (% female)	331 (32.2)	290 (32.8)	41 (28.1)	0.024
BMI	27.4 (±3.9)	27.0 (±3.7)	29.4 (±4.1)	<0.001
SBP (mmHg)	145.3 (±19.2)	145.3 (±19.5)	145.6 (±17.0)	0.002
HT (%)	571 (55.6)	467 (53.0)	104 (71.2)	0.002
FPG (mmol/l)	5.8 (5.3–6.2)	5.7 (5.3–6.2)	6.3 (6.1–6.6)	<0.001
TG (mmol/l)	1.1 (0.8–1.6)	1.1 (0.8–1.5)	1.3 (1.0–1.7)	<0.001
HDL (mmol/l)	1.3 (1.1–1.6)	1.4 (1.1–1.6)	1.2 (1.0–1.4)	<0.001
Cystatin C(mg/l)	1.05 (0.95–1.19)	1.01 (0.95–1.18)	1.08 (0.95–1.22)	0.114

BMI; body mass index, SBP; systolic blood pressure; FPG; fasting plasma glucose, HT; hypertension, TG; triglycerides, HDL; high-density lipoprotein cholesterol. Values are displayed as means (±standard deviation) or, for skewed variables, medians and interquartile (25–75) range.

### Associations of proteins with incident diabetes

In age- and sex-adjusted Cox analyses (model 1), 7 proteins were associated with incident diabetes and fulfilled the prespecified Bonferroni-corrected p-value of <5.5 × 10^−4^: paraoxonase-3 (PON3) (p = 3.3 × 10^−9^), fatty acid binding protein −4, (FABP4) (p = 9.3 × 10^−9^), plasminogen activator inhibitor 1 (PAI) (p = 4.0 × 10^−8^),insulin-like growth factor-binding protein 2 (IGFBP-2) (p = 2.9 × 10^−7^), scavenger receptor cysteine rich type 1 protein M130 (CD163) (p = 3.9 × 10^−6^), cathepsin D (CTSD) (p = 5.2 × 10^−4^) and Galectin-4 (Gal-4) (p = 5.4 × 10^−4^). (Table 3). Age- and sex adjusted Cox regression analysis examining all 91 proteins association to incident diabetes can be found in Supplemental Table 1.

**Table 3:** Cox Regression Analysis Examining Proteins relation to Incident Diabetes.

	HR (95% CI)	p-value	HR (95% CI)	p-value	HR (95% CI)	p-value
	*Model 1*		*Model 2*		*Model 3*	
PON 3	0.65 (0.56–0.75)	3.3 × 10^−9^	0.79 (0.67–0.93)	0.005	0.81 (0.69–0.96)	0.014
FABP4	1.74 (1.44–2.10)	9.3 × 10^−9^	1.46 (1.16–1.84)	0.001	1.48 (1.17–1.87)	0.001
PAI	1.70 (1.41–2–05)	4.0 × 10^−8^	1.50 (1.21–1.84)	<0.0001	1.40 (1.14–1.72)	0.001
IGFBP-2	0.66 (0.56–0.77)	2.9 × 10^−7^	0.82 (0.68–0.99)	0.039	0.89 (0.74–1.08)	0.24
CD163	1.50 (1.26–1.77)	3.0 × 10^−6^	1.34 (1.11–1.60)	0.002	1.22 (1.02–1.46)	0.029
CTSD	1.33 (1.13–1.56)	5.2 × 10^−4^	1.20 (1.00–1.43)	0.050	1.08 (0.91–1.29)	0.39
Gal-4	1.37 (1.15–1.64)	5.4 × 10^−4^	1.30 (1.08–1.56)	0.005	1.27 (1.07–1.52)	0.008

Cox regressions for incident diabetes (146 cases vs. 880 controls in the MPP) adjusted for age and sex (Model 1) and age, sex, BMI, HTN, lnTG, lnHDL lncystatin C and physical activity (Model 2) and age, sex, BMI, HTN, lnTG, lnHDL, lncystatin C, physical activity and lnglucose (Model 3). PON 3; Paraoxonase,,FABP4; fatty acid binding protein 4, PAI; Plasminogen activator inhibitor 1, IGFBP-2; Insulin-like growth factor-binding protein 2, CD163; scavenger receptor cysteine rich type 1 protein M130, CTSD; Cathepsin D, Gal-4; Galectin-4.

When further adjusting for established risk factors (model 2), all 7 proteins remained significantly associated with incident diabetes; 5 proteins (CD163, Gal-4, CTSD, PAI and FABP4) with an increased risk of diabetes and 2 proteins (PON3 and IGFBP-2) with a decreased risk for incident diabetes: **(**Table 3**)**. When further entering fasting plasma glucose (highly associated with incident diabetes; HR 1.30, 95% CI: 1.25–1.35; p = 9.1 × 10^−39^) as a covariate (model 3), the following four proteins remained significantly associated with increased risk of diabetes; PAI, Gal-4, CD163 and FABP4. Only PON3 remained significantly associated with decreased risk of diabetes (Table 3).

### Associations of proteins with prevalent diabetes

All 7 proteins associated with incident diabetes in model 1 were significantly associated (p-values < 5.5 × 10^−4^) with prevalent diabetes in a binary logistic regression model 1. However, in the fully adjusted model 3 only Gal-4 and PAI were nominally significantly associated with prevalent diabetes. **(**Table 4**)**

**Table 4:** Logistic Regression Analysis Examining Proteins relation to Prevalent Diabetes

	OR (95% CI)	p-value	OR (95% CI)	p-value	OR (95% CI)	p-value
	*Model 1*		*Model 2*		*Model 3*	
PAI	1.35 (1.22–1.50)	2.0 × 10^−8^	1.00 (0.89–1.13)	0.984	0.80 (0.67–0.95)	0.012
FABP4	1.61 (1.44–1.81)	3.6 × 10^−16^	1.16 (1.01–1.34)	0.049	1.02 (0.82–1.27)	0.847
CD163	1.58 (1.40–1.78)	8.3 × 10^−14^	1.35 (1.20–1.52)	3.5 × 10^−7^	1.17 (0.99–1.38)	0.061
Gal4	1.97 (1.76–2.22)	6.8 × 10^−30^	1.85 (1.63–2.10)	1.7 × 10^−21^	1.54 (1.29–1.84)	1.0 × 10^−6^
PON3	0.59 (0.53–0.66)	1.7 × 10^−21^	0.78 (0.45–1.35)	6.6 × 10^−5^	0.94 (0.79–1.12)	0.495
IGFBP2	0.60 (0.54–0.67)	4.2 × 10^−20^	0.77 (0.68–0.88)	1.1 × 10^−4^	1.04 (0.86–1.26)	0.672
CTSD	1.73 (1.55–1.92)	1.4 × 10^−23^	1.46 (1.30–1.65)	2.3 × 10^−10^	1.14 (0.96–1.35)	0.140

Logistic regressions for prevalent diabetes (681 cases in the MPP) adjusted for age and sex (Model 1) and age, sex, BMI, SBP, HT, TG, HDL, physical activity and cystatin C (Model 2) and age, sex, BMI, SBP, HT, TG, HDL, physical activity, cystatin C and FPG (Model 3). PAI; Plasminogen activator inhibitor 1, FABP4; fatty acid binding protein, CD163; scavenger receptor cysteine rich type 1 protein M130, Gal-4; Galectin-4, PON 3; Paraoxonase, IGFBP-2; Insulin-like growth factor-binding protein 2, CTSD; Cathepsin D.

### Harrell’s concordance index models

The basic model 1 yielded a C-index of 0.542 and an addition of any one of the 7 proteins resulted in a gain in C-statistics ranged from 4.4–10.7 percentage-units. Furthermore, an addition any one of the 7 proteins to the basic model 2 (C-index 0.692) resulted in a gain in C-statistic ranged from 0.02–1.4 percentage-units.

Finally, as compared with the basic model 3 (C-index 0.780) additions any one of the 5 proteins resulted in a gain in C-statistic that ranged from 0–0.5 percentage-units (Supplementary Table 2).

## Discussion

In this community-based sample of 1026 older individuals without known diabetes, we identified 7 proteins associated with incident diabetes. To the best of our knowledge, 3 of these associations (CTSD, Gal-4 and PON3) have not been previously reported.

### Proteins with a previously established association with incident diabetes

#### Scavenger receptor cysteine rich type 1 protein M130 (CD163)

Our findings are in line with a large prospective cohort study, which found a significantly increased risk of incident diabetes in subjects with high baseline CD163 levels^17^. CD163 is implicated in adipose tissue inflammation and may represent a glucose-independent mechanism in diabetes^17^.

#### Fatty acid binding protein, adipocyte (FABP4)

Increased FABP4 has earlier been associated with diabetes^18^. FABP4 may act as a mediator between diabetes and obesity due to its role in lipid metabolism and glucose utilization^19^.

#### Plasminogen activator inhibitor 1 (PAI-1)

A recent meta-analysis supported a link between PAI-1 and incident diabetes^20^, which is in concert with our findings that also imply the association to be glucose-independent. In addition, alleles of various single nucleotide polymorphisms (SNPs) which elevate plasma PAI-1, are individually associated with type 2 diabetes^21^, suggesting a causal relationship.

#### Insulin-like growth factor-binding protein 2 (IGFBP-2)

Inter-individual heterogeneity in endogenous IGFBP levels may influence the risk of developing type 2 diabetes^22^ and in a prospective nested case-control investigation, plasma IGFBP-2 levels were strongly and inversely associated with the risk of diabetes^23^ which is consistent with the protective effects of IGFBP-2 seen in our study.

### Proteins with a novel association with incident diabetes

#### Cathepsin D (CTSD)

A recent proteomic study showed a cross-sectional association between CTSD and prevalent insulin resistance^8^. This finding together with our finding that CTSD is associated with both prevalent and incident diabetes suggest that CTSD may have a mechanistic role in the development of diabetes and insulin resistance. The main effects of the lysosomal endopeptidase CTSD include intracellular protein turnover and extracellular matrix breakdown^24^. It has been suggested that CSTD acts a mediator between obesity and chronic adipose tissue inflammation as weight gain has shown to stimulate CTSD activity leading to adipocyte apoptosis, which is an important contributor to insulin resistance^25^. Furthermore, increased CTSD activity has in experimental studies been shown to be involved in the truncation of ApoA1 (the most abundant protein in HDL) to ApoA1Δ(1–38); a variant which is more abundant in patients with diabetes and more susceptible to oxidation^26^.

#### Galectin-4 (Gal-4)

Gal-4 is a small lectin protein expressed almost exclusively in the gastrointestinal tract and is involved in protein apical trafficking and lipid raft stabilization i.e. the transport of proteins from inside the cell to the cell membrane. One of the proteins transported from the Golgi apparatus to the apical cell membrane of the enterocyte is the protease dipeptidyl peptidase-4 (DPP-4)^27^. DPP-4’s most known effect is the inactivation of our two most abundant incretins; glucose-dependent insulinotropic polypeptide (GIP) and proglucagon-derived peptide glucagon-like peptide-1 (GLP-1)^28^. GLP-1-analogues and DPP-4-inhibitors are well-established treatments in type 2 diabetes and recently two major studies of GLP-1-analogues have shown, in addition to lowering blood glucose, a reduced risk of cardiovascular disease^29,30^ and mortality^30^. One possible explanation of our finding that Gal-4 is associated with both incident and prevalent diabetes is that increased expression of Gal-4 leads to increased activity of DPP-4 and thus reduced activity of GLP-1 and increased risk of diabetes and cardiovascular complications. Although other galectins (e.g. Gal-3^31^ and Gal −1^32^) have been associated with diabetes, no association of Gal-4 with diabetes has, to our knowledge, been reported before.

#### Paraoxonase type 3 (PON3)

PON3 is similar to paraoxonase type 1 (PON1) in activity but differs from it in substrate specificity^33^. Both PON3 and PON1 are bound to HDL and because of their similar properties as antioxidants, it is possible PON3 also plays a role in the prevention of LDL and HDL oxidation^34^. Previous studies have consistently reported that PON1 is lower in patients with diabetes compared to control subjects^35^. Although we could not find previous data regarding PON3 in plasma and subsequent risk of diabetes, there are studies that have has described lower levels of PON3 with an increased duration of diabetes and in patients with diabetes and coronary artery disease (CAD) compared to subjects with diabetes without CAD^36,37^. All these findings are in line with the diabetes protective effects of PON3 seen in our study.

#### Study limitations

Since type 2 diabetes is a multifactorial disease with a range of known risk factors contributing to its pathogenesis, these risk factors should be considered when conclusions are drawn regarding associations. Although we attempted adjustment for a heterogeneous panel of risk factors, the observational nature of this study prevents us from ruling out that other confounders may have affected the outcome of our analysis. Furthermore, we did not have the possibility for repeated or confirmatory measurements of the proteins through an additional method. Baseline HbA1c was missing in >30% of the subjects and therefor excluded which is a weakness as HbA1c is a very strong predictor for incident diabetes. There was no oral glucose tolerance test performed in these subjects. Moreover, our data was collected at a single regional center, without the option of replicating the findings although we attempted to limit the risk of false positive findings by Bonferroni correction. The original selection of the population with oversampling of groups based on glucometabolic disturbances mentioned in the *Methods* section can raise concerns how well this cohort represents the background population but the rate of incidence of diabetes in this cohort is comparable to other similar cohorts^38,39^. Furthermore, as mentioned in the *Methods* section, type of diabetes was not specified from the registries but we have assumed that the incidence of type 1 diabetes must be extremely low due to the participants′ mean age of 67.4 (±6.0) years at the baseline examination.

Lastly, although the CVD III panel is only partially directed towards metabolism, it also includes proteins associated with cardiovascular disease and inflammation and thus a more specifically designed assay towards diabetes and/or metabolism could possibly have revealed additional findings.

## Conclusion

Our study confirmed previously established associations with incident diabetes for CD163, FABP4, PAI, and IGFBP-2. Furthermore, we identified novel associations for CTSD, Gal-4 and PON3 with incident diabetes. Gal-4 and PON3 remained significantly associated with incident diabetes after adjusting for plasma glucose, implying a glucose independent association with diabetes. None of the proteins showed a substantial increase in C-index which, at present, would not warrant clinical use as a biomarker. Nevertheless, the associations of these three proteins could represent novel biological mechanisms, broadening our understanding of the complex pathogenesis of diabetes. First and foremost, our results merit replication in an independent cohort and if successful, future prospective studies to clarify their role in the possible pathogenesis of diabetes.

## Electronic supplementary material

Supplementary Table 1

Supplementary Table 2
