# Supplementary material for: Using a Targeted Proteomics Chip to Explore Pathophysiological Pathways for Incident Diabetes– The Malmö Preventive Project
Source: Sci Rep. 2019 Jan 22;9:272. doi: 10.1038/s41598-018-36512-y (PMC6342982; doi:10.1038/s41598-018-36512-y)
Supplement: Supplementary file 1 — Supplementary Table 1 [file 41598_2018_36512_MOESM1_ESM.docx]

**SUPPLEMENTARY TABLE 1.
Tracking id:** SREP-18-23292C

**Using a Targeted Proteomics Chip to Explore Pathophysiological Pathways for Incident Diabetes– The Malmö Preventive Project**

John Molvin^1,2^, Manan Pareek^3,4^, Amra Jujic^1^, Olle Melander^1,5^, Lennart Råstam^1^, Ulf Lindblad^6^, Bledar Daka^6^, Margrét Leósdóttir^1,2^, Peter M. Nilsson^1,5^, Michael H. Olsen^3,7^, Martin Magnusson^1,2,8^

*^1^Department of Clinical Sciences, Lund University, Clinical Research Center, Malmö, Sweden*

*^2^Department of Cardiology, Skåne University Hospital Malmö, Sweden*

*^3^Cardiology Section, Department of Internal Medicine, Holbæk Hospital, Holbæk, Denmark*

*^4^Brigham and Women’s Hospital Heart & Vascular Center, Harvard Medical School, Boston, Massachusetts, USA
^5^ Department of Internal Medicine, Skåne University Hospital, Malmö, Sweden
 ^6^ Institute of Medicine, Department of Public Health and Community Medicine, Sahlgrenska Academy, University of Gothenburg, Gothenburg, Sweden
^7^Centre for Individualized Medicine in Arterial Diseases (CIMA), Odense University Hospital, University of Southern Denmark, Denmark*

*^8^ Wallenberg Center for Molecular Medicine, Lund University, Sweden***Corresponding author and request for reprints:** John Molvin, MD, PhD-student. ^2^Department of Cardiology, Inga Marie Nilssons Gata 46, 2nd floor, Skåne University Hospital, SE 205 02 Malmö, Sweden. Email: johnmolvin@gmail.com Telephone: +46-40-331902, Fax: +46-40-336209

**SUPPLEMENTARY TABLE 1. Age and sex adjusted Cox Regression Analysis Examining all 91 Proteins relation to Incident Diabetes**

| **Protein** | **Hazard ratio** | **95% CI** | | **p-value** |
| --- | --- | --- | --- | --- |
|  |  | **Lower** | **Upper** |  |
| PON3 | 0,649 | 0,563 | 0,749 | 3,28E-09 |
| FABP4 | 1,737 | 1,439 | 2,097 | 9,29E-09 |
| PAI | 1,698 | 1,406 | 2,051 | 4,02E-08 |
| IGFBP2 | 0,657 | 0,560 | 0,772 | 2,90E-07 |
| CD163 | 1,496 | 1,264 | 1,772 | 2,96E-06 |
| CTSD | 1,328 | 1,131 | 1,559 | 5,18E-04 |
| Gal4 | 1,373 | 1,147 | 1,643 | 5,40E-04 |
| GDF15 | 1,349 | 1,137 | 1,601 | 6,03E-04 |
| tPA | 1,318 | 1,122 | 1,549 | 7,77E-04 |
| RARRES2 | 1,334 | 1,120 | 1,589 | 1,22E-03 |
| IL1RT2 | 1,314 | 1,109 | 1,557 | 1,62E-03 |
| LDLreceptor | 1,291 | 1,090 | 1,529 | 3,05E-03 |
| CTSZ | 1,282 | 1,083 | 1,519 | 3,92E-03 |
| PDGFsubunitA | 1,314 | 1,086 | 1,589 | 4,93E-03 |
| UPAR | 1,278 | 1,075 | 1,519 | 5,49E-03 |
| TNFR2 | 1,239 | 1,050 | 1,463 | 1,12E-02 |
| TNFR1 | 1,234 | 1,045 | 1,457 | 1,33E-02 |
| CSTB | 1,220 | 1,039 | 1,432 | 1,54E-02 |
| PI3 | 1,231 | 1,040 | 1,456 | 1,57E-02 |
| SELE | 1,238 | 1,041 | 1,472 | 1,59E-02 |
| PCSK9 | 1,229 | 1,037 | 1,457 | 1,71E-02 |
| PSPD | 1,216 | 1,024 | 1,445 | 2,55E-02 |
| IGFBP7 | 1,206 | 1,021 | 1,423 | 2,74E-02 |
| TNFRSF14 | 1,205 | 1,021 | 1,423 | 2,78E-02 |
| PLC | 1,200 | 1,013 | 1,422 | 3,47E-02 |
| JAMA | 1,183 | 1,009 | 1,388 | 3,85E-02 |
| CCL16 | 1,197 | 1,006 | 1,424 | 4,27E-02 |
| MB | 1,185 | 1,005 | 1,399 | 4,38E-02 |
| IL17RA | 1,187 | 1,001 | 1,407 | 4,84E-02 |
| TR | 1,174 | 0,989 | 1,394 | 6,64E-02 |
| TNFSF13B | 1,170 | 0,988 | 1,386 | 6,84E-02 |
| ITGB2 | 1,170 | 0,988 | 1,386 | 6,95E-02 |
| MCP1 | 1,155 | 0,986 | 1,352 | 7,44E-02 |
| MPO | 1,168 | 0,985 | 1,385 | 7,46E-02 |
| IGFBP1 | 0,867 | 0,739 | 1,019 | 8,25E-02 |
| COL1A1 | 0,874 | 0,749 | 1,019 | 8,64E-02 |
| proBNP | 1,176 | 0,975 | 1,420 | 9,04E-02 |
| Gal3 | 1,152 | 0,973 | 1,364 | 1,00E-01 |
| MMP9 | 1,149 | 0,969 | 1,362 | 1,10E-01 |
| CXCL16 | 1,140 | 0,968 | 1,343 | 1,16E-01 |
| AZU1 | 1,138 | 0,968 | 1,337 | 1,17E-01 |
| SHPS1 | 1,146 | 0,966 | 1,359 | 1,17E-01 |
| FAS | 1,128 | 0,969 | 1,313 | 1,20E-01 |
| CCL15 | 1,135 | 0,967 | 1,334 | 1,22E-01 |
| vWF | 1,134 | 0,964 | 1,334 | 1,30E-01 |
| IL18BP | 1,137 | 0,962 | 1,342 | 1,31E-01 |
| CASP3 | 1,136 | 0,961 | 1,343 | 1,35E-01 |
| PRTN3 | 1,130 | 0,959 | 1,331 | 1,45E-01 |
| TFPI | 0,885 | 0,751 | 1,043 | 1,45E-01 |
| ALCAM | 1,130 | 0,954 | 1,337 | 1,57E-01 |
| uPA | 1,126 | 0,955 | 1,328 | 1,57E-01 |
| CCL22 | 1,116 | 0,949 | 1,313 | 1,84E-01 |
| GRN | 1,114 | 0,944 | 1,314 | 2,00E-01 |
| OPG | 1,118 | 0,941 | 1,329 | 2,06E-01 |
| IL2RA | 1,108 | 0,937 | 1,311 | 2,31E-01 |
| AXL | 1,100 | 0,935 | 1,295 | 2,50E-01 |
| CCL24 | 1,100 | 0,935 | 1,295 | 2,52E-01 |
| EPHB4 | 0,909 | 0,773 | 1,071 | 2,54E-01 |
| KLK6 | 1,101 | 0,931 | 1,304 | 2,61E-01 |
| PECAM1 | 1,096 | 0,933 | 1,286 | 2,64E-01 |
| ST2 | 1,104 | 0,926 | 1,316 | 2,69E-01 |
| SPON1 | 1,096 | 0,922 | 1,301 | 2,98E-01 |
| PGLYRP1 | 1,080 | 0,916 | 1,273 | 3,59E-01 |
| TNFRSF10C | 1,081 | 0,914 | 1,279 | 3,62E-01 |
| CHI3L1 | 1,079 | 0,914 | 1,273 | 3,69E-01 |
| TFF3 | 1,081 | 0,910 | 1,286 | 3,75E-01 |
| BLMhydrolase | 1,076 | 0,914 | 1,267 | 3,81E-01 |
| TRAP | 1,068 | 0,905 | 1,262 | 4,36E-01 |
| SELP | 1,065 | 0,905 | 1,253 | 4,47E-01 |
| APN | 1,065 | 0,902 | 1,258 | 4,59E-01 |
| Notch3 | 1,061 | 0,893 | 1,261 | 5,02E-01 |
| LTBR | 1,056 | 0,895 | 1,247 | 5,18E-01 |
| MMP3 | 1,051 | 0,873 | 1,266 | 5,97E-01 |
| TIMP4 | 1,046 | 0,880 | 1,244 | 6,10E-01 |
| CDH5 | 0,959 | 0,814 | 1,128 | 6,11E-01 |
| RETN | 1,041 | 0,881 | 1,229 | 6,39E-01 |
| CPA1 | 1,036 | 0,878 | 1,221 | 6,77E-01 |
| OPN | 1,031 | 0,873 | 1,218 | 7,21E-01 |
| CNTN1 | 1,031 | 0,870 | 1,223 | 7,22E-01 |
| TLT2 | 1,030 | 0,873 | 1,215 | 7,27E-01 |
| CD93 | 0,973 | 0,825 | 1,148 | 7,45E-01 |
| ICAM2 | 0,974 | 0,827 | 1,147 | 7,52E-01 |
| CPB1 | 1,026 | 0,870 | 1,209 | 7,62E-01 |
| MMP2 | 1,021 | 0,865 | 1,207 | 8,03E-01 |
| DLK1 | 1,021 | 0,865 | 1,204 | 8,08E-01 |
| MEPE | 1,013 | 0,858 | 1,197 | 8,76E-01 |
| SCGB3A2 | 0,989 | 0,841 | 1,164 | 8,97E-01 |
| EGFR | 0,991 | 0,838 | 1,171 | 9,12E-01 |
| IL6RA | 0,995 | 0,845 | 1,171 | 9,49E-01 |
| IL1RT1 | 0,996 | 0,844 | 1,175 | 9,62E-01 |
| EpCAM | 0,996 | 0,845 | 1,174 | 9,63E-01 |
